# Accuracy assessment of implant placement with versus without a CAD/CAM surgical guide by novices versus specialists via the digital registration method: an in vitro randomized crossover study

**DOI:** 10.1186/s12903-023-03116-6

**Published:** 2023-06-27

**Authors:** Sha Li, Chun Yi, Ziyang Yu, Aozhou Wu, Yu Zhang, Ye Lin

**Affiliations:** 1 Department of Oral Implantology, Peking University School and Hospital of Stomatology & National Clinical Research Center for Oral Diseases & National Engineering Research Center of Oral Biomaterials and Digital Medical Devices & Beijing Key Laboratory of Digital Stomatology, 22 Zhongguancun South Avenue, Haidian District, 100081 Beijing, P. R. China; 2 Department of Oral Implantology, Peking University School and Hospital of Stomatology Center of Digital, Dentistry, Peking University School and Hospital of Stomatology & National Clinical Research Center for Oral Diseases & National Engineering Research Center of Oral Biomaterials and Digital Medical Devices & Beijing Key Laboratory of Digital Stomatology, Beijing, P. R. China; 3 Department of Epidemiology, Johns Hopkins Bloomberg School of Public Health, Baltimore, MD USA

**Keywords:** Dental implants, Dimensional measurement accuracies, Computer-assisted surgeries, Digital technologies, Simulation training

## Abstract

**Background:**

Many studies demonstrated that surgical guides might reduce discrepancies compared with freehand implant placement. This randomized crossover study aimed to assess the effects of approaches, practitioners’ experience and learning sequences on the accuracy of single tooth implantation via digital registration method. No similar study was found.

**Methods:**

This in vitro randomized crossover study enrolled 60 novice students (Group S) and 10 experienced instructors (Group I). Sixty students were randomly and evenly assigned to two groups (Group SA and SB). In Group SA, 30 students first performed single molar implant on a simulation model freehand (Group SAFH), and then with a CAD/CAM surgical guide (Group SASG). In Group SB, another 30 students first performed guided (Group SBSG) and then freehand (Group SBFH). Ten instructors were also divided into Group IAFH/IASG (*n* = 5) and IBSG/IBFH (*n* = 5) following the same rules. The accuracy of implant placement was assessed by the coronal and apical distance (mm) and angular (°) deviations using the digital registration method. T tests and nonparametric tests were used to compare the results among different groups of approaches, experience and sequences.

**Results:**

For students, the coronal and apical distance and the angular deviations were significantly lower in surgical guide group than freehand group in total and in learning freehand first subgroup, but for learning surgical guide first subgroup the apical distance deviation showed no significant difference between two approaches. For students, the angular deviation of freehand group was significantly lower in learning surgical guide first group than learning freehand first group.

For instructors, the coronal and apical distance and angular deviations showed no significant difference between two approaches and two sequences.

For freehand approach, the coronal and apical distance and the angular deviations were significantly higher in student group than instructor group, while not significantly different between two groups for surgical guide approach.

**Conclusions:**

For novices, using a surgical guide for the first implant placement may reduce the potential deviations compared with freehand surgery, and may reach a comparable accuracy with that of specialists. For simple single molar implantation, the surgical guide may not be significantly helpful for experienced specialists.

## Background

### Review of existing literature and the necessity of the study

Appropriate three-dimensional (3D) positioning of dental implants is a crucial factor for the long-term success of implant therapy. It ensures good implant stability, bone and soft tissue support, and optimal prosthesis design, in terms of function, aesthetics, occlusion, loading transfer and hygiene maintenance accessibility [1].

Experienced implant surgeons may achieve a relatively high accuracy in the position, depth, and angulation for simple implant placement after a long-term extensive training and practice. However, for novice practitioners, such as dental students without any previous implant placement experience, achieving predictable implant placement based on anatomical structures and the prosthetic requirements can be a great challenge [2].

Dental implant educators have been continuously seeking effective methods for assisting novice practitioners to perform implant placement competently and accurately [3]. C.M. Ardila and D. Gonzalez-Arroyave reported that dental students showed great interest in learning and utilizing the new CAD/CAM techonologies, and they recognized that their abilities improved implementing CAD/CAM techniques [4]. Higher efficacy in terms of learning skills and knowledge gained was observed and great satisfaction was expressed when the 3D systems were used by medical students. The 3D systems had a higher efficacy in helping medical students learn and acquire new skills and knowledge compared to traditional teaching methods, and gained great satisfaction by the students [5]. Traditional training methods that involve freehand implant placement on models are inadequate in providing reliable guidance for novice practitioners to achieve optimal planned positioning during implantation.

The cone beam computer tomography (CBCT) and computer-aided virtual design software are widely utilized tools that provide clinicians, particularly novice practitioners, a clear understanding of bone shape, implant selection, and placement design. Surgical templates can be designed by incorporating the superimposed CBCT data and optical surface scanning data into the guided surgery software. Numerous previous studies have shown that the use of surgical templates can significantly reduce discrepancies between planned and actual implant positions, compared to freehand implant placement [6, 7]. Tahmaseb et al. conducted a systematic review and meta-analysis of 20 clinical studies on the accuracy of partially edentulous tooth-supported surgical guides. The mean coronal and apical distance deviation and angular deviation between the actual and planned implants were 1.2 mm (1.04 mm to 1.44 mm), 1.4 mm (1.28 mm to 1.58 mm) and 3.5°(3.0° to 3.96°), respectively [8].

A previous study of our team demonstrated that the novel digital registration method yielded comparable results, and overcame the disadvantages associated with radiation exposure and artifacts when evaluating the accuracy of implant positioning, in contrast to the conventional CBCT method [9]. Comparing to the CBCT scan method, this digital registration method reduced the biological and economic cost. Thus, digital registration is suitable for the assessment of large-scale implantation accuracy.

However, no randomized crossover studies with a sufficiently large sample size were identified that further validated the digital registration method in the accuracy assessment of implant placement with versus without a CAD/CAM surgical guide. Additionally, there is a lack of analysis on the impact of different practitioners’ experience and learning sequence, particularly in the evaluation of simulation model training.

### Purpose and hypothesis

This in vitro randomized crossover study aimed to assess and compare the accuracy of single molar implant placement in a simulation model fully guided by a CAD/CAM tooth-supported surgical template versus freehand by novice dental students and experienced instructors. The study utilized a digital registration method and sought to analyze the impact of two implant placement approaches, practitioners’ experience levels, and learning sequences.

The null hypothesis (H0) of this study posited that the accuracy of implant placement (in terms of the coronal and apical distance deviation and angular deviation) would have no significant difference between two implant placement approaches (using a CAD/CAM surgical guide vs. freehand), two levels of practitioners’ experience (students vs. instructors), and two learning sequences (learning freehand first vs. surgical guide first).

## Methods

### Design and setting of the study

This study was designed as an in vitro randomized crossover study, and was carried out in Peking University School and Hospital of Stomatology.

## Sample size calculation

G*Power software (version 3.1.6.9, Germany) was used for sample size calculation, based on the results of Park et al.’s study [10]. The mean (SD) of angular deviations were 3.17 (2.35) in surgical guide group and 7.76 (3.49) in freehand group respectively [10]. Common standard deviation from the formula ${SD}_{pooled}=\sqrt{\frac{\left({n}_{1}-1\right){SD}_{1}^{2}+({n}_{2}-1){SD}_{2}^{2}}{{n}_{1}+{n}_{2}-2}}$ was 2.83. The effect size of test-control was 1.54. The significance level (α) of 0 0.05, a power (1-β) of 0.90, and an allocation ratio of 1 were used for the calculation. The required number of pairs was 10.

## Characteristics of participants and sample enrollment

Based on the sample size calculation and the actual number of students and instructors available to participate in this study, 60 final year dental students (Group S) without implant placement experience and 10 experienced implant specialists as instructors (Group I) were enrolled.

All the final year dental students enrolled had already completed their theoretical course pertaining to implant placement. Detailed implant surgical procedures were shown through live in-person demonstrations and a video displaying the drilling sequence and the optimal location, angle, and depth for implant placement. The specialist instructions and the demonstration video were available to the students throughout the process of implant surgery.

## Randomized allocation

A computer-generated simple randomization sequence with a 1:1 allocation ratio was used to randomize the 60 students into Group SA (*n* = 30) and SB (*n* = 30), and the 10 instructors into Group IA (*n* = 5) and IB (*n* = 5). The investigator conducting the randomized allocation was not involved in the planning, surgery and measurement process. The group allocation was concealed from all the enrolled students and instructors until the assignments began.

Taking the impact of learning sequence into consideration, 60 students (Group S) were randomly and evenly assigned to two groups (Group SA and SB). In Group SA, 30 students performed the single tooth implant placement on a simulation model freehand first (Group SAFH) and then performed the same procedure fully guided by a CAD/CAM surgical template (Group SASG). In Group SB, another 30 students performed fully guided approach first (Group SBSG) and then preformed freehand approach (Group SBFH).

Ten instructors were also allocated to two groups (Group IA and IB) following the same rules. In Group IA, five instructors performed the single tooth implant placement on a simulation model freehand first (Group IAFH) and then performed fully guided approach (Group IASG). In Group IB, another five instructors perfomed fully guided approach first (Group IBSG) and then freehand approach (Group IBFH).

**Fig. 1:**
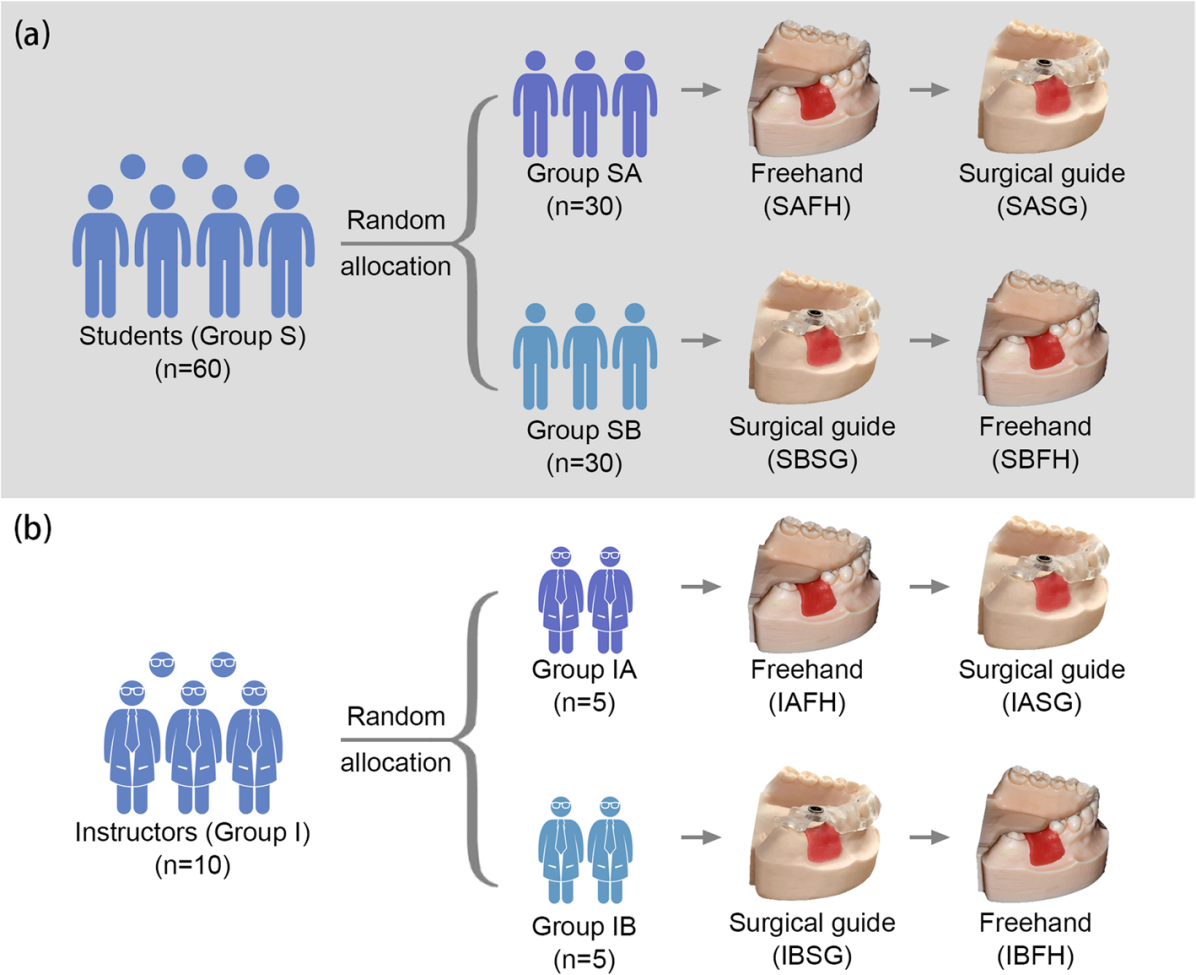
Sample size and group allocation. **a** Allocation of Student Group; **b** Allocation of Instructor Group

The sample size and group allocation were shown as Fig. 1.

### Case selection and simulation model preparation

#### Case selection

All the simulation models were duplicates and based on a real case of a 35-year-old female patient requesting implant restoration for her missing right mandibular first molar at Peking University School and Hospital of Stomatology, Department of Oral Implantology. This patient was otherwise healthy and had no contraindications to implant surgery.

**Fig. 2:**
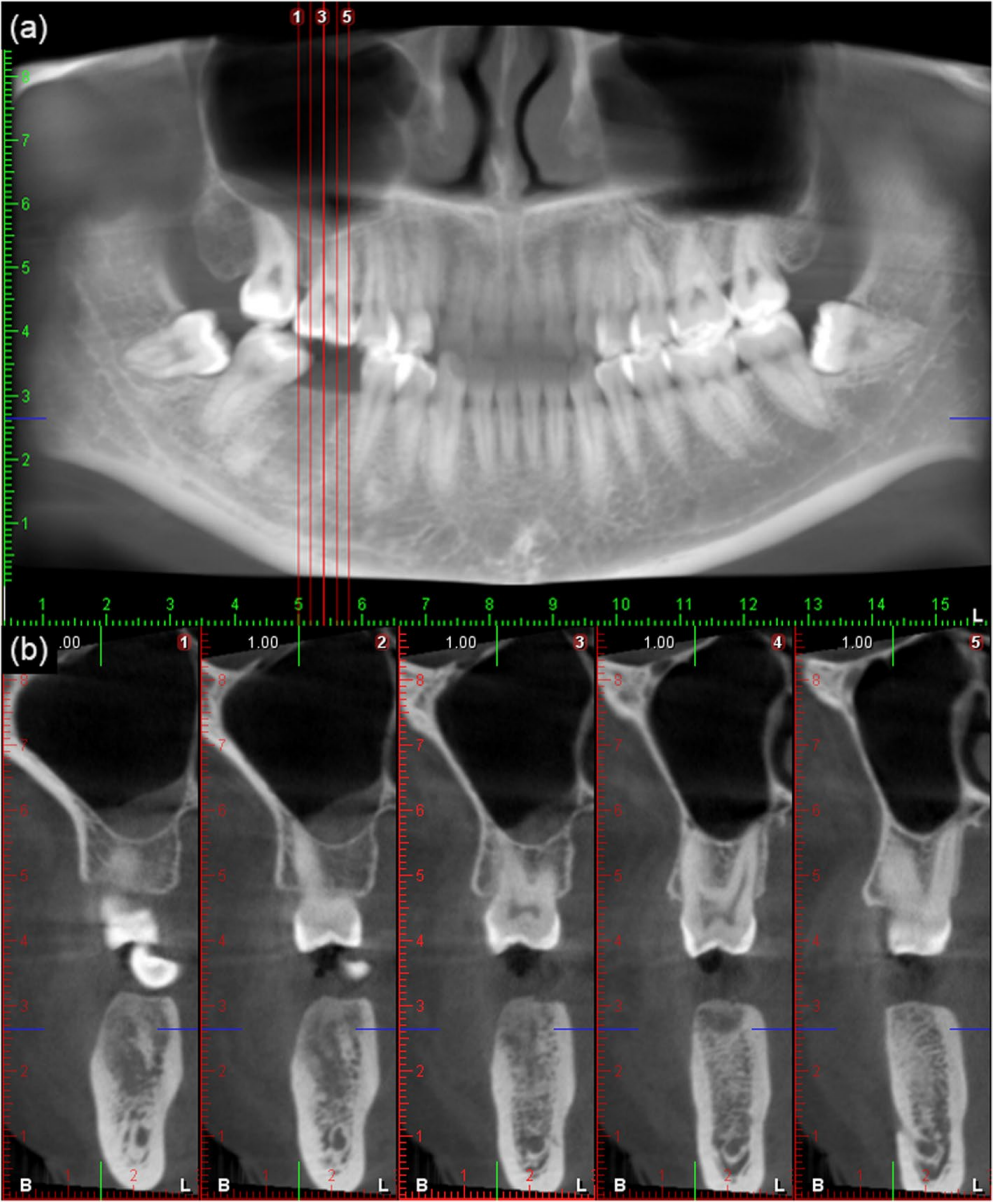
Presurgical CBCT of the patient. **a** Panoramic view; **b** Sagittal view of edentulous area

The mesiodistal space and occlusal space were sufficient for implant restoration, and the surrounding tissue was proper for implant treatment. The CBCT examination (Planmeca ProMax™ 3D scanner, Planmeca Oy, Helsinki, Finland) of the patient also showed that the 3D dimension of the alveolar ridge was adequate and the bone quality was appropriate, as shown in Fig. 2.

A written informed consent was signed for the use of her data for teaching and research purposes. All procedures related to the human participants were conducted in accordance with the 1975 Declaration of Helsinki revised in 2000 and approved by the local ethics committee (Institutional Review Board of Peking University School and Hospital of Stomatology; Approval Number: PKUSSIRB-201736075). This in vitro study protocol followed the CRIS reporting guidelines.

#### Simulation model and other material preparation

**Fig. 3:**
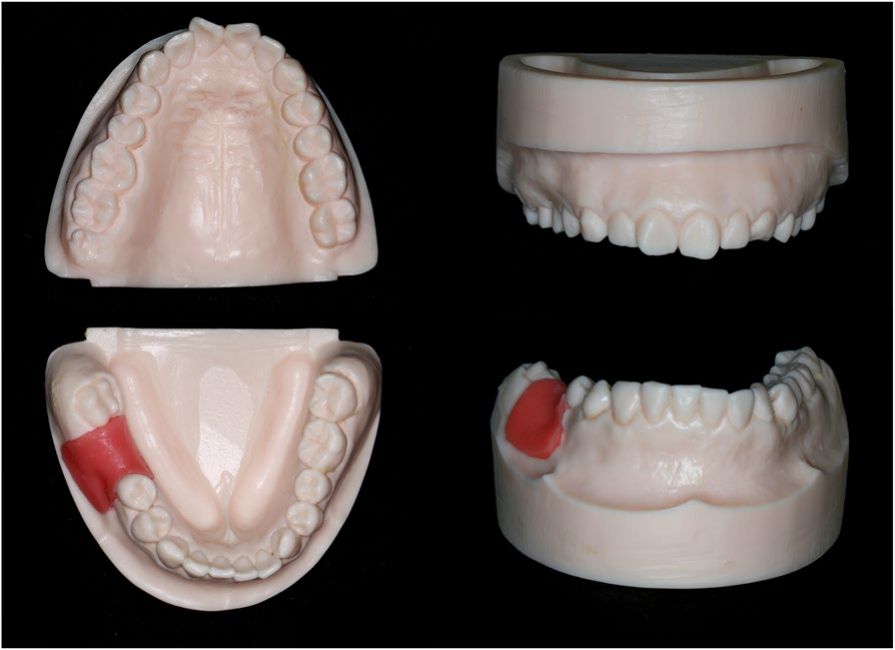
3D printed acrylic resin models (with topical artificial gingiva) of both jaws

The impressions of both jaws were taken from the patient by an experienced specialist using silicone impression material (Silagum-Light and Silagum-MixStar Putty Soft; DMG Medical Devices, Rome, Italy). The gypsum casts (Modern Materials, Die-Stone; Kulzer GmbH, Hanau, Germany) of both jaws were then poured from the impressions, and served as master models. To capture the precise details of the master models, optical scanning was performed via a highly accurate dental laboratory scanner (3Shape E4; 3Shape, Copenhagen, Denmark). As the working master model, the mandibular model was scanned three times to confirm its reproducibility. The 3D data of the model scanning was exported into STL format. A total of 140 mandibular and 35 maxillary acrylic resin models (mandibular models with topical artificial gingiva) were fabricated by 3D printers (AccuFab-C1s; SHINING 3D, Hangzhou, China) from a dental laboratory based on the STL files of the model scanning, as shown in Fig. 3. The 35 maxillary models were reutilized by Group FH/SG and Group A/B, since they were only opposite reference models without undergoing any actual procedures and changes.

### Surgical interventions

#### Surgical guide group

##### Presurgical digital design and fabrication of the template

**Fig. 4:**
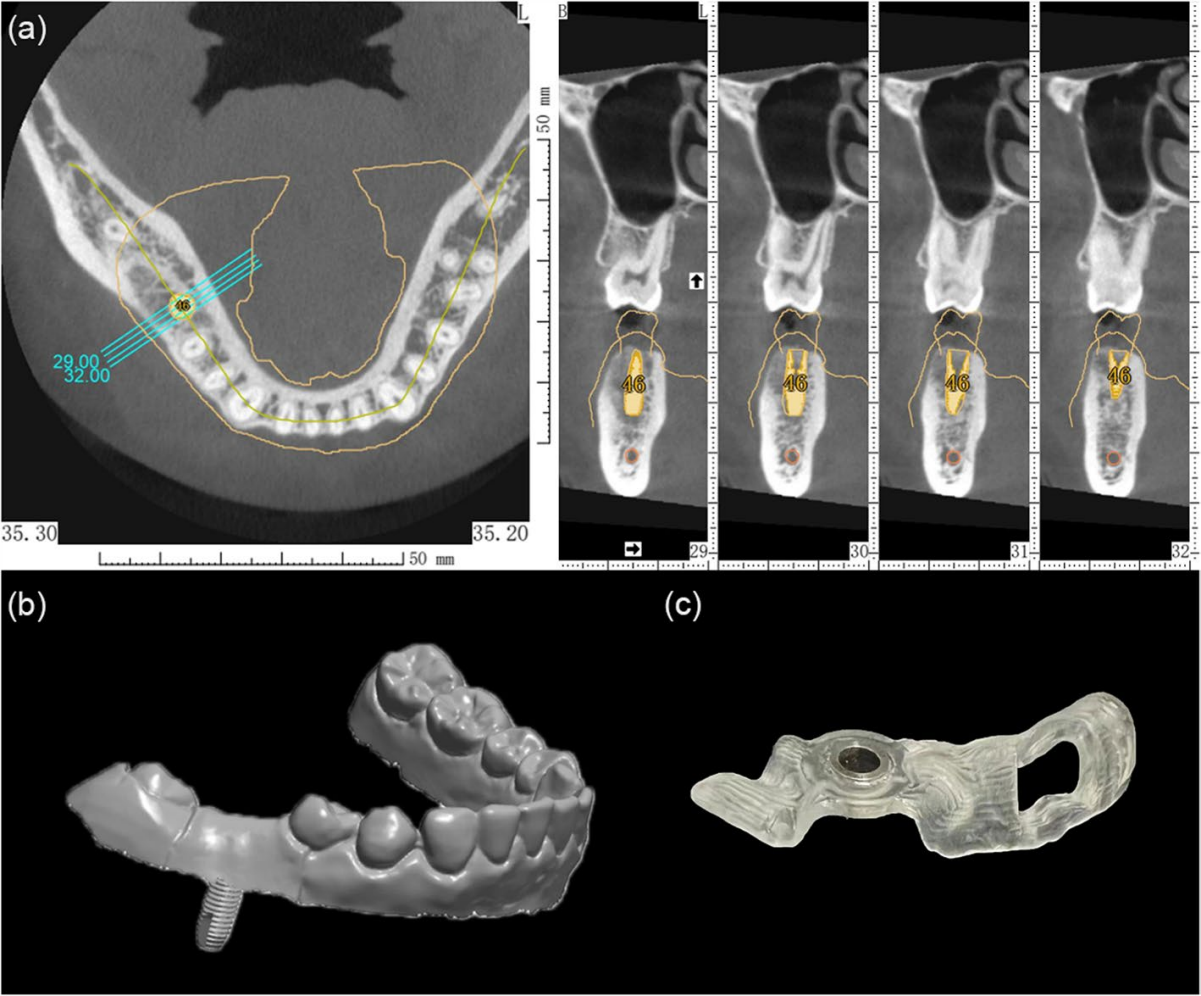
Presurgical digital design and fabrication of the surgical template. **a** Prosthetic and biological-driven digital design of implant position based on the superimposed CBCT data and optical scanning data; **b** Generation of a STL model based on presurgical design; **c** Fabrication of the CAD/CAM surgical guide

The 3D CBCT data was exported as a Digital Imaging and Communications in Medicine (DICOM) file. The CBCT data (DICOM format) along with the optical scanning data of the mandibular master model (STL format) were imported into the implant planning software (Simplant, v11.04; Dentsply Sirona, Ballaigues, Switzerland) to design the ideal implant position. The optical scanning data were aligned with the CBCT data, and a prosthetic-driven virtual set-up was created. The implant position was designed based on the virtual prosthesis and anatomical structures, by all the students and instructors individually first. The optimal implant position for the final surgical guide design was determined and approved by all the 10 instructors, as shown in Fig. 4. All the 60 students involved in the study also previsualized the planned implant position before the actual surgery.

After the implant position had been planned, a tooth-supported fully guided drilling template (CEREC Guide Bloc medi; Dentsply Sirona) was designed and sent to the milling unit (CEREC MC XL Premium; Dentsply Sirona). After cleaning and polishing of the templates, titanium sleeves were positioned into the drilling templates, as shown in Fig. 4.

##### Fully guided surgical procedures

The 3D printed acrylic resin models of both jaws were mounted to the dental dummy in the simulation laboratory. Proper seating of the tooth‐supported CAD/CAM surgical template on the model was confirmed before the surgery. The fully guided implant surgery was performed on the simulation model complying with the instructions provided by the manufacturer. The artificial gingiva was removed with a punch drill and then the implant bed was prepared following the standard drilling sequence fully guided by the template. An implant (4.2 × 13 mm; Astra Tech Implant System® OsseoSpeed® EV, Dentsply Sirona) was inserted with the drilling template in situ.

#### Freehand group

##### Presurgical planning

The implant position was only assessed and planned by the intra-oral examination (on the simulation model) and the CBCT examination, based on the mesiodistal and occlusal space, as well as the adjacent anatomical structures, estimated by individual practitioner.

##### Freehand surgical procedures

The implant surgeries were also performed on the acrylic resin simulation models mounted to the dental dummies. The only difference from the surgical guide group was performing the surgery freehand.

### Accuracy evaluation of the implant via digital registration method

The digital registration method was applied to identify the postsurgical position and assess the accuracy of each implant enrolled in this study.

The independent implant used in the surgery (4.2 × 13 mm; Astra Tech Implant System® OsseoSpeed® EV, Dentsply Sirona) was connected to a compatible scan body(AE42-SB; TruAbutment, Irvine, CA, USA), and this integrated component defined as a registration unit was scanned by a lab scanner (3Shape E4, 3Shape). The virtual 3D model of the registration unit was reconstructed by reverse engineering and saved in STL format (STL-Registration unit) [9].

Each enrolled implant inserted in the acrylic resin model was connected to the same scan body (AE42-SB; TruAbutment). Then the entire postsurgical model was optically scanned by the same lab scanner (3Shape E4; 3Shape), and scanning data of the model was also saved in STL format (STL-Actual model) [9].

The files of STL-Registration unit and STL-Actual model were imported into reverse engineering software (Geomagic Studio 2014; Geomagic, 3D Systems) and superimposed by the “best-fit alignment” function, with reference to the scan body data, which was regarded as the common region within the two STL files. The postsurgical implant position was subsequently obtained by digital registration and was exported in STL format (STL-Actual model & implant) [9].

The planned implant position data (STL-Planned model & implant) and the actual postsurgical implant position data (STL-Actual model& implant) were imported into Geomagic software (Geomagic Studio 2014), and aligned by the “best-fit alignment” function, according to the corresponding sites of the dentitions [9].

To remain only the positional relationship between the planned and actual implant positions with a clear view, the redundant parts (such as dentition and gingiva) of the models were deleted by the “selecting bounded components” function, and the scan body part was also trimmed by the “trimming with a plane” function in Geomagic software [9].

The implant coronal and apical points were labeled using the “rotation axis” function, which was automatically fitted by the software according to the contour of the implant. The intersections of the rotation axis and the implant cervix/bottom were regarded as the coronal/apical points of the implant. Points 1 and 2 were defined as the coronal and apical points of the planned implant, respectively. Points 3 and 4 were defined as the coronal and apical points of the actual implant, respectively [9].

### Outcome measurement

The Accuracy of implant placement was evaluated by three parameters:

1. linear distance deviations (mm) between the planned and actual implants at the coronal point (distance between points 1 and 3);
2. linear distance deviations (mm) between the planned and actual implants at the apical point (distance between points 2 and 4);
3. and angular deviation (°) between implant axes.

All the measurements were conducted by the same evaluating investigator who did not participate in the previous process and was blind to the group allocation, surgical procedures, and model scanning during the entire analytical stage of the study.

**Fig. 5:**
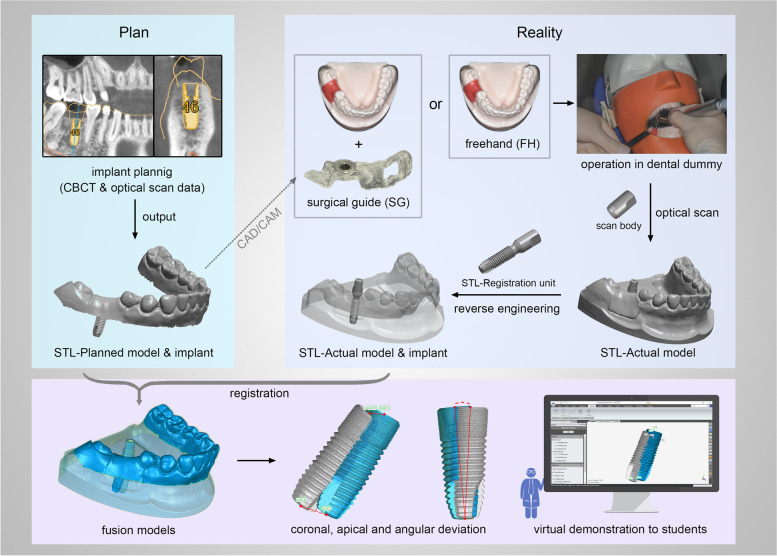
Complete workflow of the study

The complete workflow of the study was depicted in Fig. 5.

### Statistical analysis

SPSS software (version 26; IBM Corp., Armonk, NY, USA) was used for statistical analysis.

Kolmogorov–Smirnov tests (K-S test) were used to check if the results of the coronal and apical linear distance and the angular deviations of each group were in accordance with normal distribution.

Paired-samples T tests (in accordance with normal distribution) or Wilcoxon tests (not in accordance with normal distribution) were used to analyze the differences in the coronal and apical linear distance and the angular deviations between two surgery approaches (Group SG vs. FH) within the same practitioner group, i.e., Group SASG vs. SAFH, Group SBSG vs. SBFH, Group SSG vs. SFH, Group IASG vs. IAFH, Group IBSG vs. IBFH and Group ISG vs. IFH, respectively.

Independent-samples T tests (in accordance with normal distribution) or Mann–Whitney U-tests (not in accordance with normal distribution) were used to analyze the differences in the coronal and apical linear distance and the angular deviations between two learning sequences (Group A vs. B) and two groups of practitioners’ experience (Group S vs. I) with the same surgery approach, i.e., Group SASG vs. SBSG, Group SAFH vs. SBFH, Group IASG vs. IBSG, Group IAFH vs. IBFH, Group SSG vs. ISG, and Group SFH vs. IFH, respectively.

Descriptive analyses were performed for all variables.

## Results

The coronal and apical distance and the angular deviations of student group were in accordance with normal distribution in total (Group SSG and SFH) and in subgroups (Group SASG, SAFH, SBSG and SBFH). The coronal and apical distance and the angular deviations of instructor group were not in accordance with normal distribution in total (Group ISG and IFH) and in subgroups (Group IASG, IAFH, IBSG and IBFH). The corresponding statistical methods used for the comparison of different groups were shown as Tables 1, 2, 3, 4 and 5.

**Table 1:** The comparison of the coronal distance deviations between two surgery approaches (Group SG vs. FH)

Group	SG		FH		Statistical method	Difference	95% CI	*P*
	n	Mean ± SD (mm)	n	Mean ± SD (mm)				
SA	30	0.61 ± 0.29	30	1.00 ± 0.56	paired-samples T test	-0.39	-0.62 ~ -0.17	0.001^**^
SB	30	0.67 ± 0.40	30	1.10 ± 0.48		-0.43	-0.67 ~ -0.19	0.001^**^
S	60	0.64 ± 0.35	60	1.05 ± 0.52		-0.41	-0.57 ~ -0.25	< 0.001^***^
IA	5	0.48 ± 0.16	5	0.64 ± 0.13	Wilcoxon test	-0.16	/	0.080
IB	5	0.56 ± 0.45	5	0.56 ± 0.30		0.00	/	0.893
I	10	0.52 ± 0.32	10	0.60 ± 0.22		-0.08	/	0.285

^**^p < 0.01

^***^p < 0.001

**Table 2:** The comparison of the apical distance deviations between two surgery approaches (Group SG vs. FH)

Group	SG		FH		Statistical method	Difference	95% CI	*P*
	n	Mean ± SD (mm)	n	Mean ± SD (mm)				
SA	30	1.04 ± 0.48	30	1.79 ± 0.70	paired-samples T test	-0.75	-1.07 ~ -0.41	< 0.001^***^
SB	30	1.14 ± 0.61	30	1.48 ± 0.61		-0.34	-0.68 ~ 0.01	0.053
S	60	1.09 ± 0.55	60	1.63 ± 0.67		-0.54	-0.77 ~ -0.30	< 0.001^***^
IA	5	1.04 ± 0.40	5	0.87 ± 0.25	Wilcoxon test	0.17	/	0.686
IB	5	0.71 ± 0.41	5	0.69 ± 0.36		0.02	/	1.000
I	10	0.88 ± 0.42	10	0.78 ± 0.31		0.10	/	0.646

^***^p < 0.001

**Table 3:** The Comparison of angular deviations between two surgery approaches (Group SG vs. FH)

Group	SG		FH		Statistical method	Difference	95% CI	*P*
	n	Mean ± SD (°)	n	Mean ± SD (°)				
SA	30	2.74 ± 1.55	30	6.79 ± 2.89	paired-samples T test	-4.05	-5.32 ~ -2.79	< 0.001^***^
SB	30	3.05 ± 1.81	30	4.38 ± 1.73		-1.33	-2.35 ~ -0.31	0.012^*^
S	60	2.89 ± 1.68	60	5.58 ± 2.66		-2.69	-3.56 ~ -1.83	< 0.001^***^
IA	5	3.11 ± 1.45	5	2.55 ± 1.33	Wilcoxon test	0.56	/	0.500
IB	5	1.66 ± 1.22	5	2.32 ± 1.09		-0.66	/	0.345
I	10	2.38 ± 1.48	10	2.44 ± 1.15		-0.06	/	0.959

^*^p < 0.05

^***^p < 0.001

**Table 4:** The comparison results between two learning sequences (Group A vs. B)

Group	Method	Measurement	Mean ± SD		Difference	Statistical method	95% CI	*P*
			**A**	**B**				
Students	SG	Coronal distance deviation	0.61 ± 0.29	0.67 ± 0.40	-0.06	independent-samples T test	-0.24 ~ 0.12	0.512
		Apical distance deviation	1.04 ± 0.48	1.14 ± 0.61	-0.10		-0.38 ~ 0.19	0.504
		Angular deviation	2.74 ± 1.55	3.05 ± 1.81	-0.31		-1.18 ~ 0.56	0.484
	FH	Coronal distance deviation	1.00 ± 0.56	1.10 ± 0.48	-0.10	independent-samples T test	-0.37 ~ 0.17	0.479
		Apical distance deviation	1.79 ± 0.70	1.48 ± 0.61	0.31		-0.03 ~ 0.65	0.073
		Angular deviation	6.79 ± 2.89	4.38 ± 1.73	2.41		1.19 ~ 3.65	< 0.001^***^
Instructors	SG	Coronal distance deviation	0.48 ± 0.16	0.56 ± 0.45	-0.07	Mann–Whitney U test	/	0.834
		Apical distance deviation	1.04 ± 0.40	0.71 ± 0.41	0.32		/	0.295
		Angular deviation	3.11 ± 1.45	1.66 ± 1.22	1.46		/	0.175
	FH	Coronal distance deviation	0.64 ± 0.13	0.56 ± 0.30	0.08	Mann–Whitney U test	/	0.346
		Apical distance deviation	0.87 ± 0.25	0.69 ± 0.36	0.18		/	0.602
		Angular deviation	2.55 ± 1.33	2.32 ± 1.09	0.23		/	0.917

^***^p < 0.001

**Table 5:** The Comparison results between two groups of practitioners’ experience (Group S vs. I)

Method	Measurement	Mean ± SD		Difference	Statistical method	*P*
		Group S	Group I			
SG	Coronal distance deviation	0.64 ± 0.35	0.52 ± 0.32	0.12	Mann–Whitney U test	0.383
	Apical distance deviation	1.09 ± 0.55	0.88 ± 0.42	0.21		0.347
	Angular deviation	2.89 ± 1.68	2.38 ± 1.48	0.51		0.460
FH	Coronal distance deviation	1.05 ± 0.52	0.60 ± 0.22	0.45	Mann–Whitney U test	0.006^**^
	Apical distance deviation	1.63 ± 0.67	0.78 ± 0.31	0.86		< 0.001^***^
	Angular deviation	5.58 ± 2.66	2.44 ± 1.15	3.14		< 0.001^***^

^**^p < 0.01

^***^p < 0.001

**Fig. 6:**
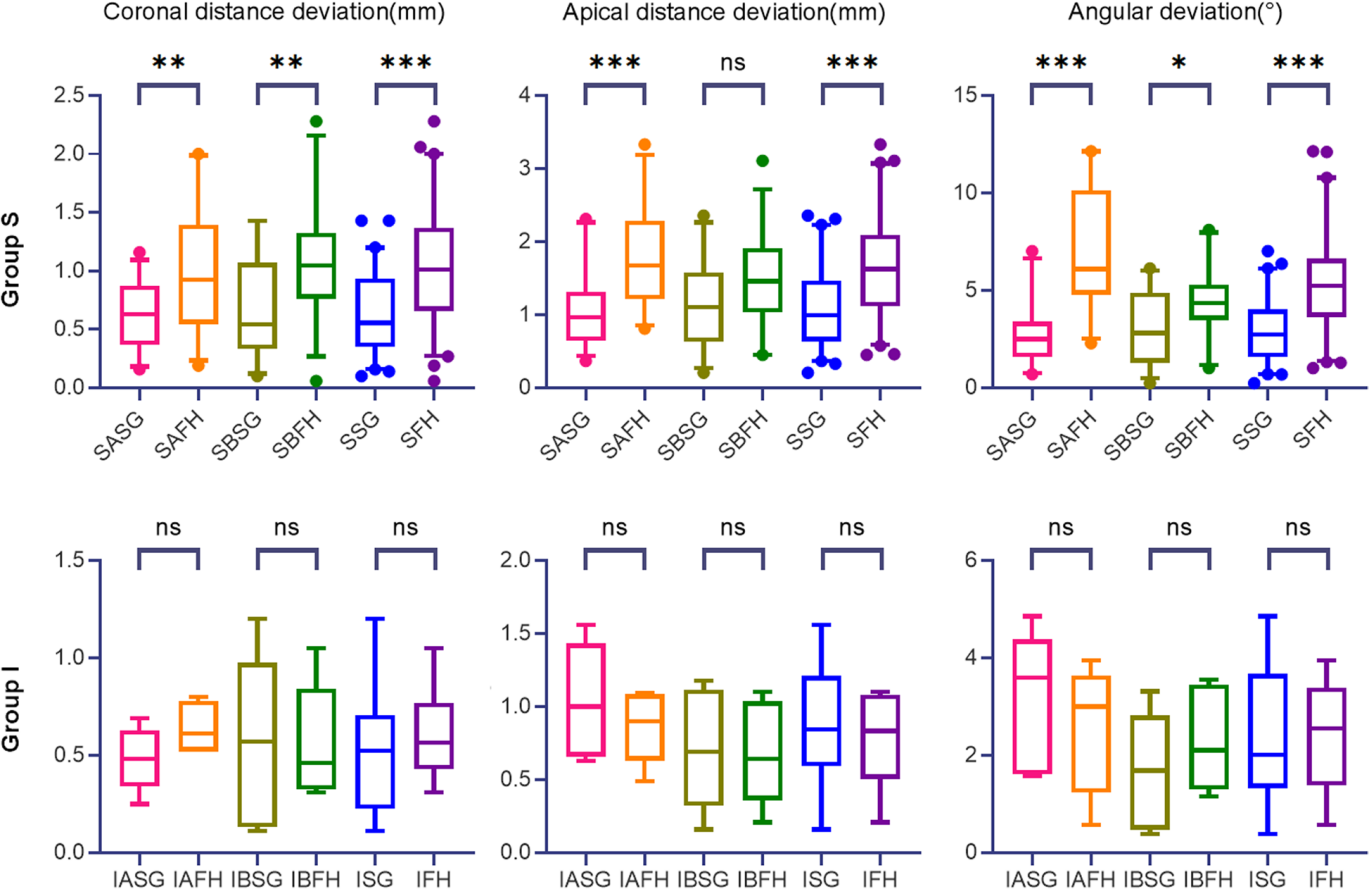
The comparison results between two surgery approaches (Group SG vs. FH)

The descriptive and comparison results of coronal and apical linear distance and the angular deviations between two surgery approaches (Group SG vs. FH) within the same practitioner group, i.e., Group SASG vs. SAFH, Group SBSG vs. SBFH, Group SSG vs. SFH, Group IASG vs. IAFH, Group IBSG vs. IBFH and Group ISG vs. IFH, are shown in Tables 1, 2 and 3 and Fig. 6 respectively.

The coronal and apical distance and the angular deviations between planned and actual implant position were significantly lower in Group SSG (0.64 ± 0.35 mm,1.09 ± 0.55 mm and 2.89 ± 1.68° respectively) than in Group SFH (1.05 ± 0.52 mm,1.63 ± 0.67 mm and 5.58 ± 2.66° respectively) in total (difference:-0.41 mm,-0.54 mm, and -2.69 mm; 95%CI: [-0.57 ~ -0.25], [-0.77 ~ -0.30] and [-3.56 ~ -1.83], respectively; *p* < 0.05) and in subgroup SA (*p* < 0.05). Therefore, for two surgery approaches, the null hypothesis was rejected in Group S and Group SA.

The coronal distance and angular deviations were significantly lower in SBSG than SBFH (*p* < 0.05), while the apical distance deviation showed no significant difference between two groups (*p* > 0.05). Therefore, for two surgery approaches, the null hypothesis was rejected in terms of coronal distance and angular deviations in Group SB, and was accepted in terms of apical distance deviation in Group SB.

The coronal and apical distance and the angular deviations showed no significant difference between Group ISG (0.52 ± 0.32 mm, 0.88 ± 0.42 mm and 2.38 ± 1.48°, respectively) and IFH (0.60 ± 0.22 mm, 0.78 ± 0.31 mm and 2.44 ± 1.15°, respectively) in total and in subgroup IA and IB respectively (*p* > 0.05). Therefore, for two surgery approaches, the null hypothesis was accepted in Group I, IA and IB.

**Fig. 7:**
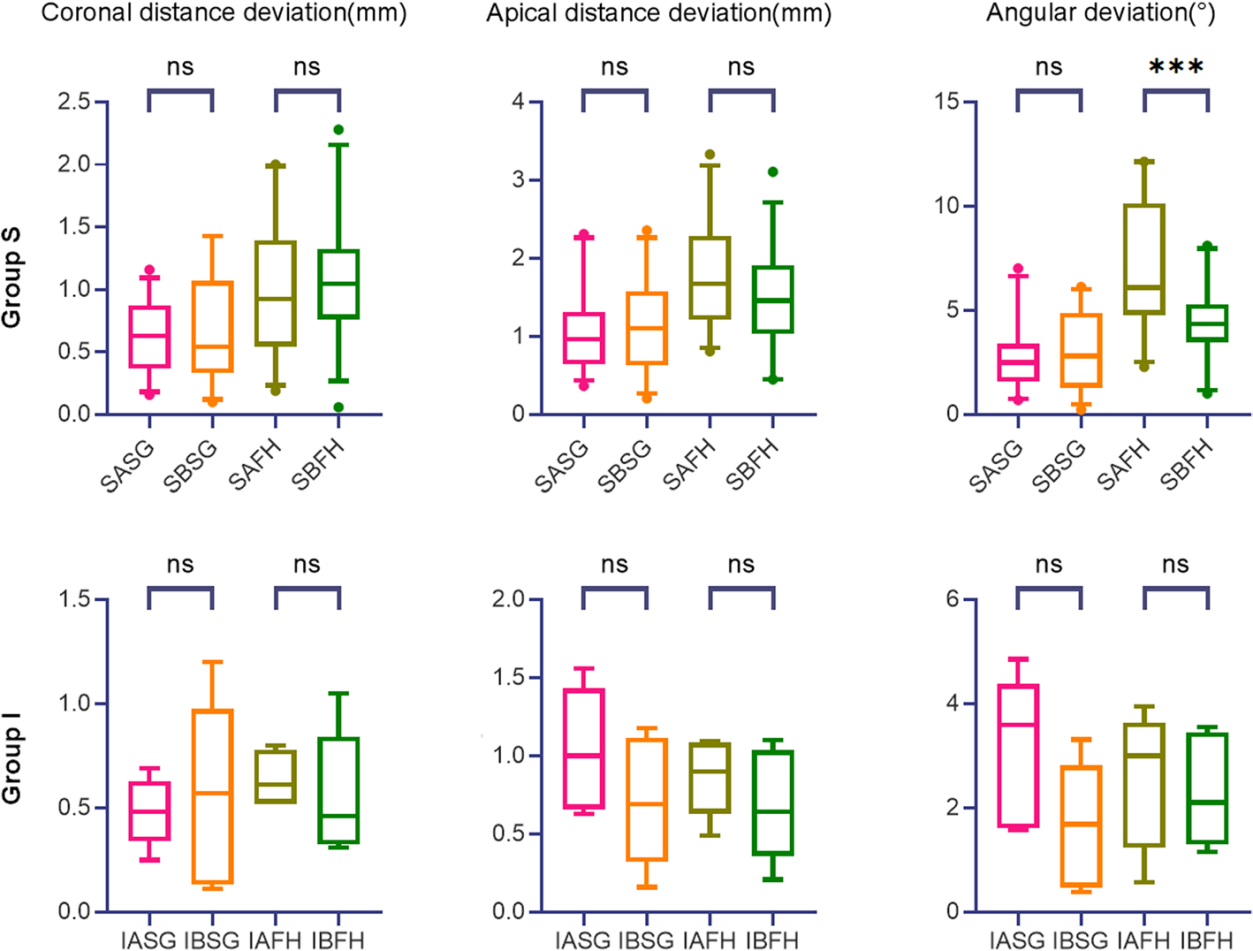
The comparison results between two learning sequences (Group A vs. B)

The comparison of coronal and apical linear distance and the angular deviations between two learning sequence (Group A vs. B) i.e., Group SASG vs. SBSG, Group SAFH vs. SBFH, Group IASG vs. IBSG, Group IAFH vs. IBFH, are shown in Table 4 and Fig. 7 respectively.

The coronal and apical distance deviation showed no significant difference between Group SASG vs. SBSG and Group SAFH vs. SBFH (*p* > 0.05). The angular deviation was significantly lower in SBFH than SAFH (*p* < 0.05), and not significantly different between SASG and SBSG (*p* > 0.05). Therefore, for two learning sequences, the null hypothesis was accepted in terms of coronal distance and apical deviations in Group SSG and SFH, and in terms of angular deviation in Group SSG. The null hypothesis was rejected in terms of angular deviation in Group SFH.

The coronal and apical distance and the angular deviations also showed no significant difference between Group IASG vs. IBSG and Group IAFH vs. IBFH (*p* > 0.05). Therefore, for two learning sequences, the null hypothesis was accepted in Group ISG and IFH.

**Fig. 8:**
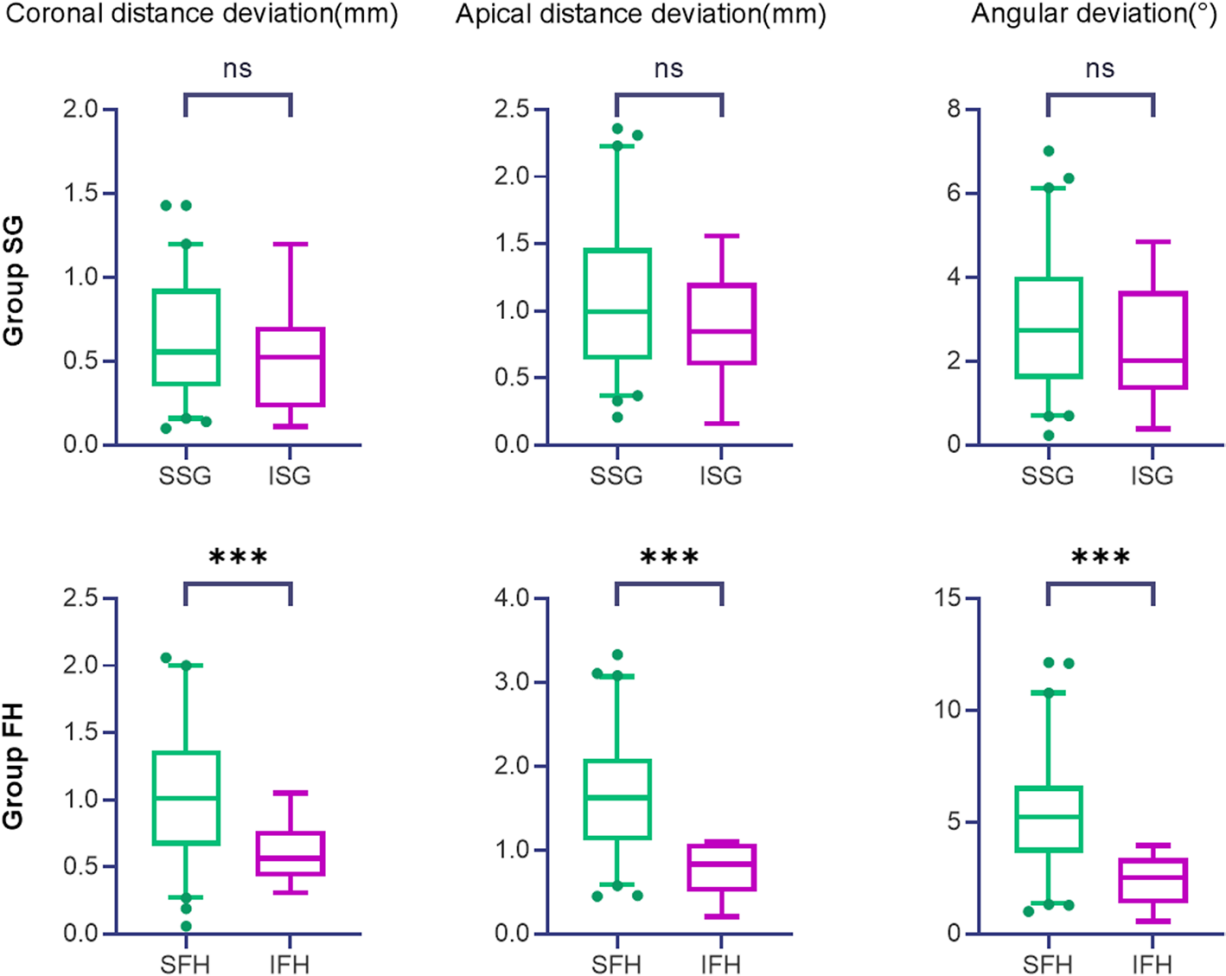
The comparison results between two groups of practitioners’ experience (Group S vs. I)

The comparison of coronal and apical linear distance and the angular deviations between two practitioners’ experience (Group S vs. I) i.e., Group SSG vs. ISG, and Group SFH vs. IFH, are shown in Table 5 and Fig. 8 respectively.

The coronal and apical distance and the angular deviations were significantly higher in Group SFH than IFH (*p* < 0.05), while not significantly different between Group SSG and ISG (*p* > 0.05). Therefore, for two levels of practitioners’ experience, the null hypothesis was rejected in Group FH, and accepted in Group SG.

## Discussion

This in vitro randomized crossover study compared the accuracy of the simple single molar implant placement in a simulation model fully guided versus freehand by novice students and experienced instructors, via the digital registration method, and analyzed the effects of two implant placement approaches, practitioner’s experience and learning sequence. The results suggested that for novice students, using a CAD/CAM surgical guide may significantly reduce the coronal and apical distance and angular deviation compared with freehand surgery, and may reach a comparable accuracy with that of experienced specialists. While for experienced specialists, the accuracy of simple single molar implant placement showed no significant difference between guided and freehand surgery. Therefore, in general, the null hypothesis was rejected for student group, but was accepted for instructor group.

### Surgical guides may improve the accuracy of implantation for novice practitioners

As this study demonstrated, the coronal and apical distance and the angular deviations between planned and actual implant position were significantly lower in Group SSG than Group SFH in total and in subgroup SA. The coronal distance and angular deviations were significantly lower in SBSG than SBFH, while the apical distance deviation showed no significant difference between two groups.

For novice practitioners, despite that adequate theoretical curricula might provide them with the basic principles of implant placement, it may still be demanding and challenging to get a visualized image and accurate guidance on the position, depth, and angulation of implant placement. Traditional training methods using freehand implant placement cannot provide reliable and predictable guidance on the optimal planned position for dental students without previous implant surgical experience, resulting in low accuracy and lacking of confidence.

There are 2 types of computer-guided implant systems currently: static guide and dynamic navigation [11]. The digital static guide is a stereolithographic implant surgical template fabricated by computer aided design (CAD) and computer-aided manufacture (CAM) technology, based on the anatomical data collected by CBCT and digital scanning. This CAD/CAM template can provide not only accurate guidance for angle or position of implant placement but also depth control with the sleeve stops of the drills in a special drill kit for safety protection. The computer-generated guide has been reported to be more accurate than conventional simple surgical guides [12, 13]. With the assistance of a CAD/CAM surgical guide, the esthetic and functional effects are desirable for edentulous cases, and the obvious deviation was dramatically reduced during positioning and drilling procedures especially for beginners [10].

### Surgical guide may not be significantly helpful for experienced specialists in simple cases

Based on the results of this study, the coronal and apical distance and the angular deviations showed no significant difference between Group ISG and IFH in total and in subgroup IA and IB.

Currently, it remains controversial whether it is more beneficial for dental implantation to use a surgical template than the traditional freehand approach in consideration of different situations. The critical review by Pozzi A. et al. suggested that there was limited evidence supporting the clinical advantage of using computer-guided surgery compared to conventional freehand implant placement for the treatment of single-tooth gap [14].

Nevertheless, a systematic review and meta-analysis by Chen et al. reported that with the technology of computer-aided surgical template, implant placement may be more accurate than freehand operation [6]. A study by Behneke A et al. also demonstrated that using a CAD/CAM surgical guide in implant placement allowed a more accurate implementation of the virtual plan than freehand insertion or freehand final drilling [15]. This study reported that surgical guides for single-tooth gaps allowed implant placement as planned with a median discrepancy of 0.16 and 0.34 mm at the implant coronal and apical level, respectively.

For most clinical situations, free-hand implantation with CBCT aiding can receive a clinically desirable position without significant error affecting subsequent prosthetics and functions. For relatively complicated cases, including inadequate alveolar bone quantity (vertical and horizontal bone defects) and limited bone available in maxillary anterior area, low bone density, multiple implant sites, implant sites near vital and complex anatomical structure (e.g., nerves or sinuses), the difficulties and risks of the implant surgeries may be dramatically increased. The surgical guides are strongly recommended in these conditions to provide good assistance and security for surgeons including very experienced ones. The self-tapping feature of the implant may result in more variation in accuracy, especially in situations with soft bone and areas with fenestration or dehiscence of the bone.

There are five main factors that may presumably influence the overall outcome: type of jaw (maxilla/ mandible), type of edentulous site (one single-tooth gap/interrupted gaps of multiple single-units/continuous partially edentulous gap of multiple units/ free-end edentulous /full-arch edentulous), type of template support (tooth-/bone-/mucosa-supported), type of guided surgery (fully guided placement/partially guided / freehand placement) and the surgical technique (flapless/open flap) [14]. As Behneke et al. reported, the linear deviation at the apical point of the implants was significantly higher in the maxillae than the mandibles (0.50 vs 0.40 mm, *P* = 0.033), while no significant differences were found for the linear deviation at the coronal point or the angular deviation [15]. As Ersoy et al. reported, the coronal and apical linear deviation and angular deviation were significantly lower in single tooth gap group (0.74 ± 0.40 mm, 1.66 ± 0.28 mm, and 3.71 ± 0.93° respectively) compared to free-end Kennedy Class I or II partially edentulous group (1.23 ± 0.67 mm, 1.59 ± 0.74 mm, and 4.78 ± 1.86° respectively) [16]. A systematic review by D’haese et al. indicated that the coronal and apical distance deviation and angular deviation ( 0.87 ± 0.40 mm, 0.95 ± 0.60 mm and 2.94° respectively) of tooth-supported guides were significantly lower compared with mucosal- and bone-supported guides [17]. A study by Younes et al. demonstrated that the fully guided surgery showed the significantly highest accuracy, while the freehand surgery showed the lowest accuracy among the three groups, and the pilot-drill guided surgery could be advantageous in certain situations where a fully guided surgery were contraindicated. Behneke et al. reported that the coronal linear deviation of implant surgeries with tooth-supported guides was significantly higher (*p* = 0.027) in flapless group than open flap group, while no significant differences was found for apical deviation and angular deviation. Erosy et al. reported that no significant difference in accuracy of guided implant surgeries for the open flap procedure versus the flapless procedure for completely or partially edentulous patients [16].

Meanwhile, several factors leading to inaccuracy of surgical guides required extra attention during each step of the workflow: presence of debris in the drilled hole preventing the implant from reaching its final position, resilience of mucosal tissues, setting of the radiological gray values during segmentation, improper seating of the template and deformation of the guide during surgery.

### Practitioners’ experience may affect the accuracy of freehand implant surgery other than guided surgery

As showed in this study, the coronal and apical distance and the angular deviations were significantly higher in Group SFH than IFH, while not significantly different between Group SSG and ISG.

The surgeon’s experience is a determinant for the accuracy of ideal implantation, particularly when implants are placed freehand without any surgical template or navigation system. The results of this study were in accordance with several studies which implicated that experience was a decided factor for implantation success, whereas others showed no significance with the assistance of the guided system [10, 18, 19].

An in vitro study by Park et al. demonstrated that the use of a CAD/CAM surgical guide reduced discrepancies among operators performing implant surgery regardless of their level of experience, and the differences of the anterior–posterior implant sites in the molar areas did not affect the accuracy of implant placement [10]. This study reported that the coronal and depth deviations were significantly higher in the inexperienced surgeon compared with the experienced surgeon in freehand group. In contrast, there was no significant difference in any of the measurements between inexperienced and experienced surgeons with the assistance of a CAD/CAM surgical guide [10]. This suggested that the surgical guide may facilitate better results by dentists who have insufficient experience performing implant surgery.

### Learning sequence may slightly affect the novice practitioners but not experienced ones

This study found that the coronal and apical distance and the angular deviations showed no significant difference between Group IASG vs. IBSG and Group IAFH vs. IBFH. The coronal and apical distance deviation showed no significant difference between Group SASG vs. SBSG and Group SAFH vs. SBFH. The angular deviation was significantly lower in SBFH than SAFH, and not significantly different between SASG and SBSG.

Implant placement in a precisely planned position requires abundant experience. Thus, a learning curve is inevitable.

For novice students, performing implant placement may have a skill acquisition and memory retention effect even after a washout period of a few days. For Group B, performing the surgical guide approach first may still achieve a relatively good accuracy even at first try due to the template guidance. This initial experience gaining of implant bed preparation and insertion, the familiarity of the drilling sequence and the hand feeling of the bone quality, might benefit the later freehand surgery to some extent. On the contrary, for Group A, the initial trial was freehand implant placement without any physical guidance nor clinical experience, thus leading to the slightly higher angular deviation compared with the accuracy of freehand implant placement in Group B. The crossover design of this study could just identify and analyze the effect of learning sequence.

### Digital registration method may be advantageous in accuracy evaluation of implant placement

The majority of previous studies used the radiographic methods to evaluate the accuracy of implant placement [20–22]. However in this study, the novel digital registration was applied based on a recent published study from our team [9]. This study introduced and validated the novel digital registration method which showed comparable results compared with the conventional CBCT method. The disadvantages of CBCT method include radiation exposure, artifacts caused by metal implants, and high cost [23–26]. Digital registration avoids the need for postsurgical radiographic examination, thereby reducing radiation exposure and other associated problems (e.g., image distortion, deformation, and artifacts). Comparing with the CBCT scan method, this digital registration method reduced the biological and economic cost. Thus, digital registration is suitable for large-scale clinical research like this particular study with large sample size.

Since the CBCT data was acquired at a voxel resolution of 0.2 mm, it was impossible to achieve superimposition precision better than 0.2 mm using conventional CBCT methods [27, 28]. A recent systematic review by Marco Cicciù et al. reported that compared to the conventional analogical impression methods, the digital optical impression methods had a comparable accuracy, and their discrepancy with reality was clinically acceptable [29]. Once the optical scanner has captured and registered the images of the implant's scan body and its adjacent teeth, the CAD software can precisely position the implant within the virtual model via algorithms [30]. The accuracy level of the lab scanner (3Shape E4, 3Shape, ISO 12836) is 4 μm according to the manufacturer’s report, which suggests that the digital registration method may provide better superior compared to the conventional CBCT approach [9]. In addition, opting for an intra-oral scanner instead of a lab scanner could potentially compromise the accuracy of this method, since the precision and trueness of intra-oral scanners tend to decrease as the scanned quadrants increase [29, 31]. However, study has demonstrated that there showed no significant difference in the dental implant location obtained via an intra-oral scanner versus an extra-oral (lab) scanner [29, 32].

The accuracy of implant surgery with and without a surgical guide was evaluated by superimposing the implant planning data and postsurgical data by digital registration method in four steps [9]. First, a virtual registration unit that included an implant replica and a scan body, was constructed based on a reverse engineering process [9]. Second, a postsurgical optical scan of the dentition was obtained with the scan body [9]. Third, the relative positions of the postsurgical implant and the adjacent dentition were identified in the first registration, which was regarded as the common region of the registration unit and the dentition scan data [9]. Fourth, a second registration was performed, which involved superimposition of the first registration data and planned implant position data according to the corresponding sites of dentition [9]. The 3D positional relationship between the planned and actual implant data was obtained after the superimposed data files had been trimmed [9].

### Fully guided implantation approach and accuracy evaluation by digital registration method may be promising for dental student simulation training

As an innovated alternative to conventional teaching methodology, computer-aided learning (CAL) and virtual simulation (VS) training have been integrated in dental education [33]. Several studies showed that the simulation of real cases and patients could effectively integrate basic science relevance, prepare for clinical problem solving, and teach new clinical content and other necessary elements in the graduate, postgraduate and continuing dental education [33–37]. In consistent with this new education philosophy, the simulation implant placement training presented in this study integrated real patient data, which had to be handled like true clinical cases. Such a problem-based learning (PBL) environment greatly promoted the development of a more motivating, pragmatic and enjoyable approach to dental implantology preclinical education according to students’ feedbacks. In addition, the more resemblance between the training workflow and the actual workflow setting, the better the performance in future clinical practice can be expected. In contrast to pure computer simulations and virtual learning facilities, a real drilling procedure with and without a surgical template was implemented and the only difference between treatment of a real patient and training on the dummy was the surgery (drilling) of the dental acrylic models instead of the real patient’s hard and soft tissues. To mimic the situation in the patient as closely as possible, proper aseptic protocols, including gloves and masks were used.

Implant placement training includes all steps in the implant surgery. The final evaluation of the accuracy of implant placement also provided the instructors with a more objective and convenient approach for teaching effect assessment. The students may also gain a more visualized and clear understanding of the ideal implant positions and angle, and how that was related to the good bone and soft tissue support, optimal prosthesis design, aesthetics, occlusion, loading transfer and access for hygiene maintenance. The visualized interface and the trimming and hiding functions of the software were also advantageous for instructors to explain in details and different view angles to the students for each case how to improve the accuracy.

The adoption of real case-based learning method and simulated fully guided surgery as shown in this study as part of dental implantology training curriculum, may promisingly improve the trainee’s development, increase their confidence in the beginning of their implantology profession and minimize the leap between laboratory and clinic.

### Limitations and future perspectives

This study was performed in vitro utilizing simulated models and dental dummies. Despite of the advancements in materials to mimic more realistic artificial gingiva and alveolar bone, the simulation model in a dental dummy was still unable to perfectly simulate real surgical conditions as human or animal cadavers. Besides, the high costs of surgical guide manufacturing, the demand for accuracy in laboratory production and patients’ desires could limit its application in training programs and clinical practice.

In addition, the sample size of the instructors’ group was relatively small compared to that of the students’ group in this study, due to the fact of limited availability of experienced instructors. A larger sample size of experienced practitioner group and a multi-center trial may improve the reliability and effectiveness of the study.

Moreover, this study only compared the accuracy of implantation between the static surgical guide approach and freehand approach by practitioners with different experience. A more comprehensive comparison among static guide, dynamic navigation and freehand approach by novices and specialists, in terms of the accuracy, time, cost and learning curve are still required. The efficiency and efficacy assessment of the digital registration method in clinical practice is also needed in the future.

## Conclusions

For novice practitioners, using a CAD/CAM surgical guide for the first implant placement may significantly reduce the potential distance and angular deviations compared with freehand surgery, and may reach a comparable accuracy with that of specialists. For simple single molar implantation, the surgical guide may not be significantly helpful for experienced specialists. The digital registration method showed predictable advantages and promising potential in the large-scale implantation accuracy evaluation. Further studies in vivo, a comparison between static guide and dynamic navigation by novices and specialists, as well as the efficiency and efficacy assessment of the digital registration method in clinical practice are still required in the future.

## Data Availability

All essential data is presented in the manuscript. The step-by-step datasets and images during the current research are available from the corresponding author on reasonable request.

## Abbreviations

CAD / CAM: Computer Aided Design / Computer Aided Manufacturing
CAL: Computer Aided Learning
CBCT: Cone Beam Computer Tomography
CRIS: Checklist for Reporting In: vitro Studies
DICOM: Digital Imaging and Communications in Medicine
Group I: Group Instructors
Group IA: Group Instructors—sequence A
Group IB: Group Instructors—sequence B
Group IFH: Group Instructors for Freehand
Group ISG: Group Instructors for Surgical Guide
Group IAFH: Group Instructors—sequence A for Freehand
Group IASG: Group Instructors—sequence A for Surgical Guide
Group IBFH: Group Instructors—sequence B for Freehand
Group IBSG: Group Instructors—sequence B for Surgical Guide
Group S: Group Students
Group SA: Group Students—sequence A
Group SB: Group Students—sequence B
Group SFH: Group Students for Freehand
Group SSG: Group Students for Surgical Guide
Group SAFH: Group Students—sequence A for Freehand
Group SASG: Group Students—sequence A for Surgical Guide
Group SBFH: Group Students—sequence B for Freehand
Group SBSG: Group Students—sequence B for Surgical Guide
K: S test-Kolmogorov–Smirnov tests
PBL: Problem-Based Learning
SD: Standard Deviation
STL: Standard Tessellation Language
VS: Virtual Simulation
